# Local Anæsthesia in General Medicine and Surgery

**Published:** 1886-08

**Authors:** 


LOCAL ANÆSTHESIA IN GENERAL MEDICINE AND SURGERY. Being the Practical Application of the Author's Recent Discoveries. By J. Leonard Carnnig, M. D., formerly Resident Assistant Physician to the Hudson River State Hospital for the Insane, Member of the Medical Society of the County of New York, Fellow New York Academy of Medicine, of the Physicians' Mutual Aid Association, of the New York Neurological Society, etc.

This little *brochure* is divided into two parts. In part first a short historical account of the various anæsthetic agents is given. Part second is devoted to an exposition of the methods of producing local anæsthesia, methods for the most part original with the author. It is rather curious to read the admission on the part of the writer that Crawford W. Long, of Georgia, was the first to use ether in surgical operations, yet in the next breath according the honor of its discovery to W. T. G. Morton, who used the agent four years later.

